# Prognostic Value of Inducible Nitric Oxide Synthase (iNOS) in Human Cancer: A Systematic Review and Meta-Analysis

**DOI:** 10.1155/2019/6304851

**Published:** 2019-06-04

**Authors:** Wenbiao Liao, Tao Ye, Haoran Liu

**Affiliations:** ^1^Department of Urology, Renmin Hospital of Wuhan University, Wuhan 430030, China; ^2^Department of Urology, Tongji Hospital, Tongji Medical College, Huazhong University of Science and Technology, Wuhan 430030, China; ^3^Hubei Institute of Urology, Wuhan 430030, China

## Abstract

**Background:**

Inducible nitric oxide synthase (iNOS) is confirmed to regulate the production of nitric oxide (NO) when cells are exposed to external stimulus. Recent publications revealed that overexpression of iNOS predicted poor clinical outcomes for patients with malignant cancers, e.g., gastric, bladder, and colorectal cancers; however, several studies reported no obvious relationship between iNOS expression and prognosis of solid tumors. The aim of our study was to investigate the pooled effect of the prognostic value of iNOS expression.

**Materials and Methods:**

We performed a systematic search of PubMed, Web of Science, and Embase databases up to January 15, 2019. The concerned outcomes of interest included overall survival (OS), cancer-special survival (CSS), and recurrence-free survival (RFS).

**Results:**

Fourteen studies with 1,758 patients were included in this meta-analysis, and we reached the conclusion that increased iNOS expression was significantly associated with worse OS (HR: 1.89, 95% CI: 1.57 - 2.28, p ≤ 0.001), worse CSS (HR: 3.13, 95% CI: 1.88 - 5.20, p ≤ 0.001), and worse RFS (HR: 2.16, 95% CI: 1.29 - 3.62, p = 0.003) in solid tumors. Furthermore, the subgroup analysis identified the significant relationship of high iNOS expression with poor OS in gastric cancer. No obvious publication bias was detected by Begg's tests.

**Conclusion:**

In summary, the results drawn in our meta-analysis demonstrated that elevated expression of iNOS had a significant association with poor survival in human cancer. iNOS might serve as a promising predictive biomarker of prognosis in cancer patients, and well-designed prospective studies are further needed to substantiate the prognostic value of iNOS.

## 1. Introduction

Malignant tumors have become a major public health problem around the world, causing great burdens on patients, families, and society. Based on the evaluation report of International Agency for Research on Cancer, approximately 18.1 million new cancer patients and 9.6 million cancer-related deaths occurred in 2018 [1]; moreover, the number of incidence and mortality is still trending upward. Although great advances have been made in the field of cancer therapy due to the development of science and technology, the prognosis of patients with malignancy is still poor, especially for those with late-stage tumor or distant metastasis [2, 3].

Nitric oxide synthase (NOS) is the critical player regulating the conversion of amino acid L-arginine to endogenous nitric oxide (NO) [4]. NO is a small and short-lived gas molecule required for a variety of physiological processes including immune responses, neurotransmission, and vasodilation. The function of NO in cancer is complicated, because it could promote and inhibit the progression of tumor depending on different conditions. High NO level may result in tumor cell apoptosis, but low level induce cell proliferation by stimulating angiogenesis [5].

The production of NO is regulated by NOSs family, including neuronal NOS (nNOS), inducible NOS (iNOS), and endothelial NOS (eNOS), whereas nNOS and eNOS are expressed in several certain types of cells and release a relatively small amount of NO; however, iNOS is an isoform induced by inflammatory stimuli and synthesizes a higher level of NO in chronic inflammation condition [6]. Therefore, iNOS is mainly responsible for the increased production of NO. Considering the complicated roles of NO in the initiation and development of cancer, numerous studies have also explored the specific role of iNOS in various human cancers. Recently, accumulated evidence indicated that the elevated expression of iNOS was significantly associated with the angiogenesis, chemotherapeutic resistance, metastasis, and immune resistance in some malignant tumors, e.g., colorectal cancer, breast cancer, bladder cancer, gastric cancer, and melanoma [7–10]. In addition, several research studies also reported that iNOS expression had a correlation with unfavorable prognosis in human cancers [11–13], but there were still many controversies surrounding the prognostic value of iNOS in tumor [14, 15]. Due to the noncomprehensive analysis for survival data in a single study, we performed an integrated meta-analysis to investigate the prognostic value of iNOS by pooling the results of multiple studies and to support the role of the protein as a promising prognostic biomarker.

## 2. Materials and Methods

### 2.1. Publication Search

Systematic literature searches of PubMed, Web of Science, and Embase databases (up to January 15, 2019) were carried out using the combination of following keywords: “inducible nitric oxide synthase” OR “iNOS” OR “NOS2”, “cancer” OR “tumor” OR “carcinoma” OR “neoplasm”, and “prognosis” OR “survival” OR “outcome”. The reference lists of relevant studies were also scanned manually for further potentially eligible articles.

### 2.2. Study Selection Criteria

To reach a convincing result in our meta-analysis, included studies had to (a) evaluate the relationship between iNOS expression and prognosis in any type of cancer; (b) directly provide hazard ratios (HRs) and corresponding 95% confidence intervals (CIs) of multivariate analyses for concerned outcome endpoints; (c) categorize cancer patients into two groups based on “high/positive” and “low/negative” iNOS expression.

Exclusion criteria included (a) literature published as reviews, letters, comments, or case reports; (b) non-human or non-English studies; (c) research studies without sufficient data for analyses.

### 2.3. Data Extraction and Quality Assessment

Two investigators independently reviewed each identified study and conducted the data extraction using a previously designed form. Any disagreement was resolved by discussions. The following data were abstracted from each included article: first author; publication year; region of origin; duration of research; sample size; cancer type; age of the patients; detection method of iNOS expression; cut-off value; follow-up period; and HRs with corresponding 95% CIs of concerned outcome endpoints (including overall survival (OS), cancer-specific survival (CSS), and recurrence-free survival (RFS)). The HRs and their 95% CIs were directly identified from the multivariate analysis in each eligible article. Besides, the Newcastle-Ottawa Scale (NOS) was applied to determine the quality of the included studies with a score ranging from 0 to 9 [16]. The study with NOS score > 6 was regarded as a study with high methodological quality.

### 2.4. Statistical Analysis

The data extracted from each eligible study were pooled to assess the strength of the association between iNOS expression and survival outcomes in human solid cancers by meta-analysis. Combined HRs with their 95% CIs for these concerned endpoints (OS, CSS, and RFS) were calculated. Subgroup analysis was conducted when more than two studies existed in each subgroup. Heterogeneity across publications was determined using the chi-square based Q test and I^2^ test. P-value < 0.1 in combination with I^2^ > 50% indicated significant heterogeneity, and a random-effect model would be applied to merge the HRs. Otherwise, a fixed-effect model should be used. Begg's linear regression test was performed to identify the possible publication bias. A sensitivity analysis was employed to check the stability of the results in our study. All the statistical analyses were conducted using the Stata 12.0 software (StatCorp, College Station, TX, USA). P-value < 0.05 indicated statistical significance for all tests except for heterogeneity analysis.

## 3. Results

### 3.1. Study Selection and Characteristics

**Figure 1:**
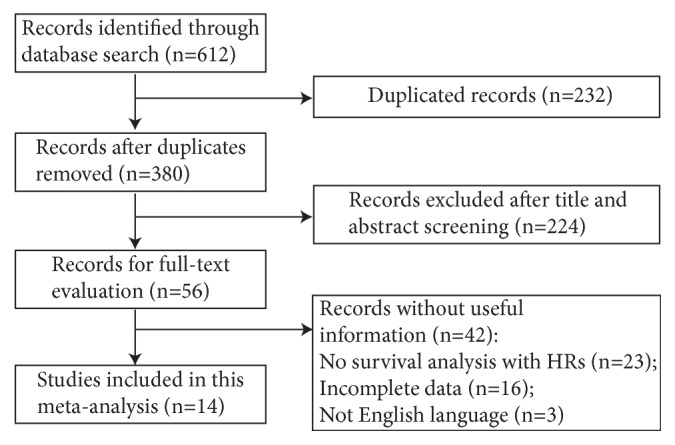
Flow diagram of the study selection process.

As shown in Figure 1, literature searches of PubMed, Web of Science, and Embase databases resulted in the inclusion of 612 publications via various combinations of previously described keywords. After excluding the duplicate records, 380 papers were assessed. For the remaining papers, 224 were excluded after reviewing the titles and abstracts. Additional 42 records were eliminated after the full-text evaluation for the absence of useful information: 23 for no survival analysis with HRs; 16 for incomplete data; and 3 for not English language. Ultimately, 14 published studies were elected for our meta-analysis [11–13, 17–27].

The main characteristics of the 14 included publications were summarized in Table 1. All the articles were published between 2002 and 2018. The sample size of individual study ranged from 63 to 248, and our meta-analysis eventually included 1,758 patients. Eight of the research studies enrolled over 100 cancer patients each, and 11 different cancer types were recorded. The expression levels of iNOS in all studies were measured by immunohistochemistry (IHC), but the cut-off values were different. As for disease outcomes, eight studies focused on OS, five on CSS, and two on RFS. The HRs with their 95% CIs were directly extracted from all the eligible studies. All the articles were considered highly qualified assessed by Newcastle-Ottawa Scale with a score > 6 in each study.

### 3.2. Meta-Analysis of OS

**Figure 2:**
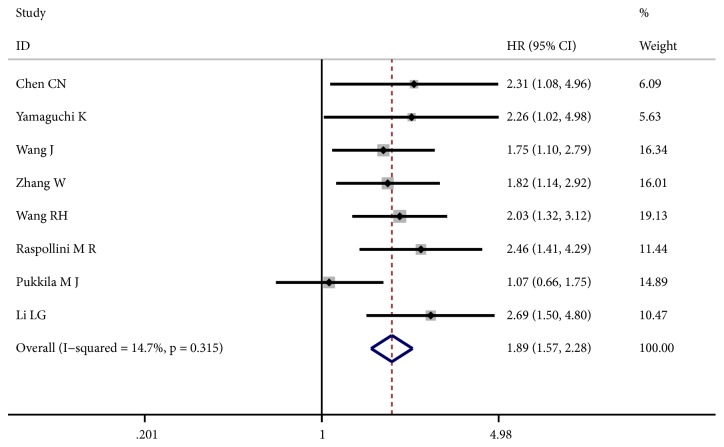
Forest plot of the effect of iNOS status on overall survival (OS) in solid tumors.

**Figure 3:**
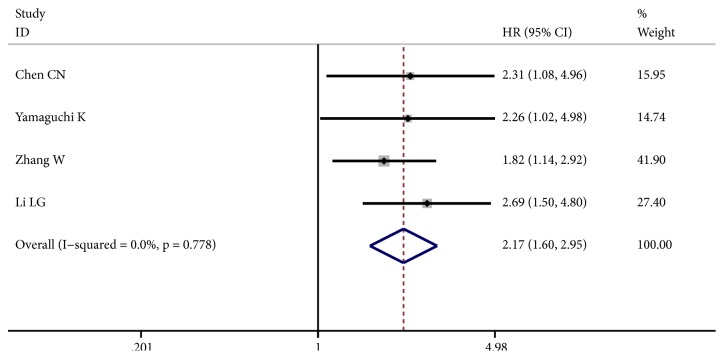
Forest plot of the effect of iNOS status on overall survival (OS) in gastric cancer.

Eight articles containing 884 participants were involved in OS of our study. As shown in Figure 2, no obvious heterogeneity was noted among the included studies (I^2^ = 14.7%, p = 0.315). The fixed-effect model was applied to calculate the combined HRs and their 95% CIs. The results indicated that patients with increased iNOS expression were significantly correlated with shorter OS (HR: 1.89, 95% CI: 1.57 - 2.28, p ≤ 0.001). Moreover, we conducted a subgroup meta-analysis on the basis of four studies which reported HRs on gastric cancer. The pooled HRs revealed a significant association between iNOS overexpression and unfavorable prognosis in gastric cancer (HR: 2.17; 95% CI: 1.60 - 2.95, p ≤ 0.001; fixed-effect) (Figure 3).

### 3.3. Meta-Analysis of CSS and RFS

**Figure 4:**
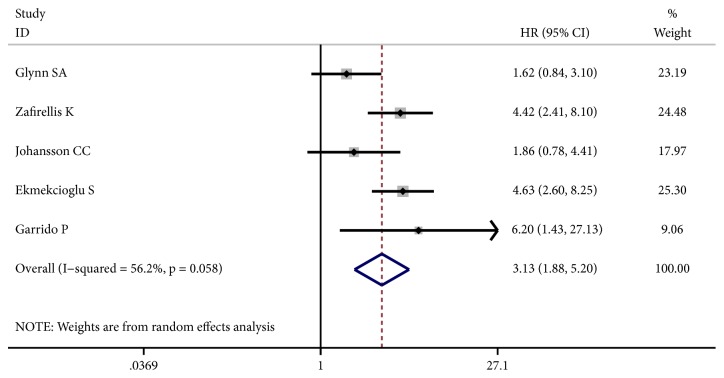
Forest plot of the effect of iNOS status on cancer-specific survival (CSS) in solid tumors.

**Figure 5:**
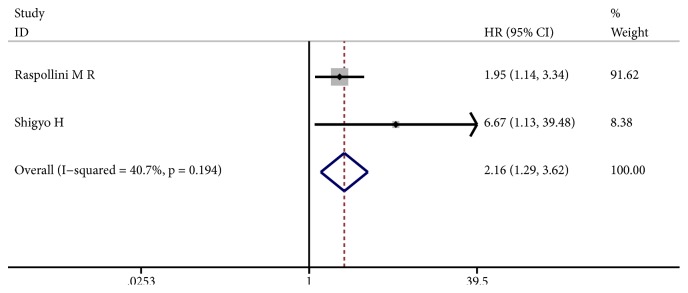
Forest plot of the effect of iNOS status on recurrence-free survival (RFS) in solid tumors.

Five studies presented HRs for CSS. Considering the evidence of significant heterogeneity (I^2^ = 56.2%, p = 0.058), a random-effect model was used. The pooled HR was 3.13 (95% CI: 1.88 - 5.20, p ≤ 0.001) (Figure 4), demonstrating that elevated iNOS expression was predictive of reduced CSS in solid tumors. Similarly, the meta-analysis of RFS including two studies suggested that patients with high iNOS expression were subjected to significantly shorter RFS (HR: 2.16, 95% CI: 1.29 - 3.62, p = 0.003) (Figure 5). The fixed-effect model was used because of no obvious heterogeneity between studies (I^2^ = 40.7%, p = 0.194).

### 3.4. Publication Bias and Sensitivity Analysis

**Figure 6:**
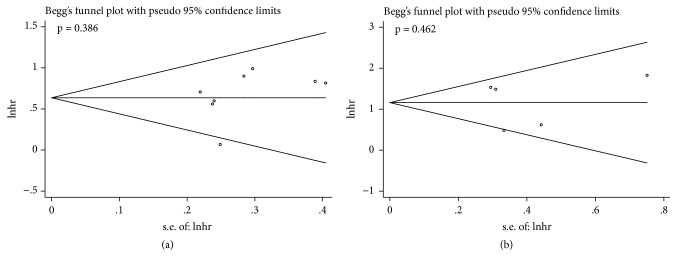
Begg's funnel plots for the studies involved in the meta-analysis. (a) Overall survival. (b) Cancer-specific survival.

**Figure 7:**
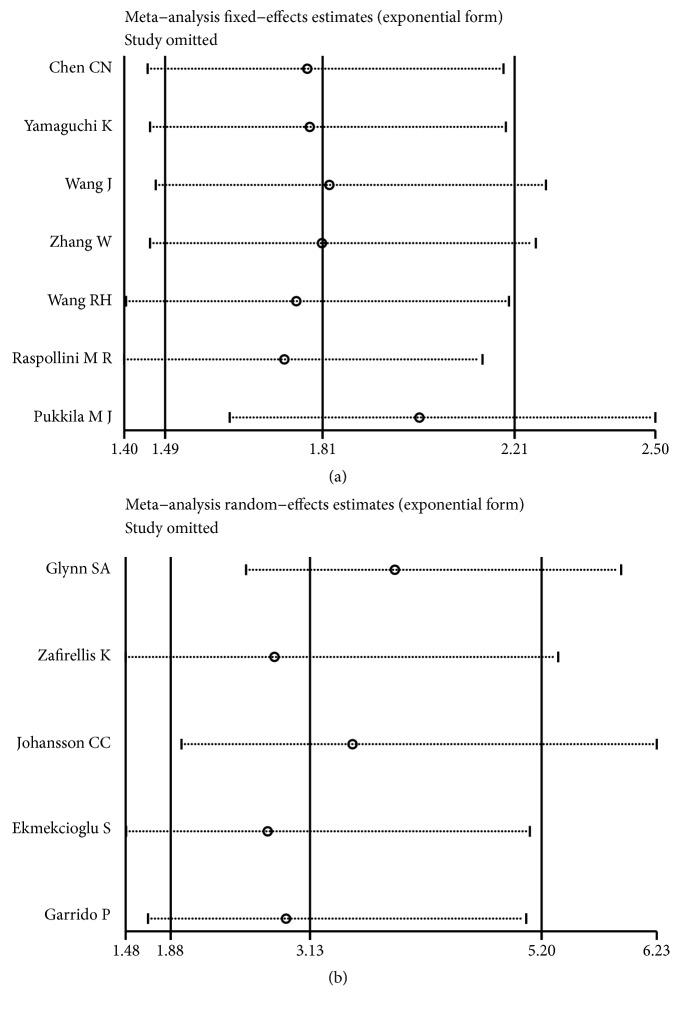
Sensitivity analysis of the meta-analysis. (a) Overall survival. (b) Cancer-specific survival.

The publication bias for the meta-analyses of OS and CSS was analyzed by Begg's tests, and the p-values were 0.386 and 0.462 (Figure 6), respectively, indicating that no significant publication bias was observed in these analyses. Furthermore, sensitivity analyses were performed, respectively, to determine the outcome stability in our meta-analysis. No individual study could control the final results, and our meta-analysis was considered stable and credible (Figure 7).

## 4. Discussion

Recently, numerous studies have reported that increased iNOS expression was involved in tumor progression and could predict unfavorable prognosis in human cancer. The prognostic role of iNOS overexpression in solid tumors has been explored by lots of clinical studies. However, most of them were unable to reach a comprehensive conclusion due to the relatively small sample size in one single study. Our present meta-analysis may be the first complete review of the published research studies evaluating the relevance between iNOS expression and prognosis of many types of human cancer.

We scientifically analyzed the survival data of 1,758 cancer patients from 14 different studies. Eleven types of human cancer were incorporated to explore the prognostic value of iNOS for solid tumors, including gastric cancer (GC), breast cancer, pancreatic cancer (PC), colorectal cancer (CRC), hepatocellular carcinoma (HCC), uveal melanoma, ovarian cancer (OC), melanoma, bladder cancer, hypopharyngeal squamous cell carcinoma (HSCC), and laryngeal squamous cell carcinoma (LSCC). The meta-analysis eventually demonstrated that iNOS overexpression could serve as a prognostic predictor for solid tumors, with the results of shorter OS (HR: 1.89, 95% CI: 1.57 - 2.28, p ≤ 0.001), shorter CSS (HR: 3.13, 95% CI: 1.88 - 5.20, p ≤ 0.001), and shorter RFS (HR: 2.16, 95% CI: 1.29 - 3.62, p = 0.003). In addition, subgroup analysis also revealed a significant relationship of high iNOS expression with poor OS in gastric cancer. These results all supported that iNOS had significant predictive value for the poor clinical outcomes of human cancers, especially gastric cancer.

Generally, the underlying mechanism of iNOS in the progression of various cancers remained unclear. The major function of iNOS was to regulate the production of NO, both of them were involved in maintaining the intracellular physiology process, and their anomalous change would cause the disruption of normal homeostasis of cells [28]. The aberrant iNOS expression has been described in many types of cancers, including prostate [29], bladder [30], breast [18], and colorectal cancers [20]. Moreover, the signaling way of iNOS/NO was confirmed to be a critical player in the progression of human cancer, and both anti- and pro-tumor functions have been published.

Many publications have indicated that the interaction of iNOS with p53, whose wide-type was a major tumor suppressor, regulated the process of tumor development. Garrido et al. reported that iNOS was involved in the activation of epidermal growth factor receptor (EGFR) and the accumulation of p53 mutation in breast cancer cell lines; in addition, iNOS overexpression was proven to correlate with disease recurrence, distant metastasis, and reduced CSS in breast cancer patients [11]. Yang Lan and his colleagues revealed that higher expression level of iNOS was observed in advanced stage oral squamous cell carcinoma (OSCC) patients with decreased survival rate, and p53 expression increased significantly after iNOS knockdown in OSCC cells [31]. A recent finding indicated that iNOS-synthesized NO displayed a distinctive feature of cancer stem cells (CSCs) which exhibited self-renewal capacity, and iNOS/NO could promote Notch1 activation via TACE/ADAM17 signal way in liver cancer stem cells (LCSCs), finally resulting in a more aggressive tumor phenotype [22]. It was known that matrix metalloproteinase-9 (MMP-9) played an essential role in cancer invasion and metastasis by degrading native type IV collagen, which usually functioned as a major structural component of basement membranes. Sun et al. reported that NO produced by iNOS could enhance the expression of MMP-9 and therefore contribute to the angiogenesis, invasion, and metastasis in HCC [32].

On the other hand, several in vitro and in vivo studies also revealed the antitumor potential of overexpressing iNOS on cancer cell kinetics. For instance, overexpression of iNOS on colorectal cancer cell lines SNU-1040 and HCT-116 via gene transfer induced a large amount of NO production and then led to an enhanced effect of radiation-induced apoptosis [33]. In a xenograft mouse model of pancreatic cancer, cancer cells with iNOS upregulation did not form solid tumors or have metastases [34]. The large synthesis of NO caused by iNOS overexpression might be an explanation for these effects.

However, several limitations also existed in our meta-analysis. First, only three electronic databases (PubMed, Web of Science, and Embase) were searched for eligible studies, so as to overlook articles written in non-English, which probably caused a potential of language bias. Second, the sample size in several reports was relatively small, potentially increasing the heterogeneity. Third, the cut-off values determining high/positive and low/negative iNOS expression by IHC method varied across studies, and it could result in some discrepancy in this meta-analysis. Considering that we extracted survival data in HRs with 95% CIs directly from the articles, the results were relatively stable and credible. In summary, the results derived from our meta-analysis indicated that overexpression of iNOS could be used as a predictor for unfavorable prognosis in human cancers and significantly correlate with poor OS of gastric cancer. In the future, further prospective studies of different cancer types with more participants are needed to validate the prognostic role of iNOS expression in various types of solid tumors.

## Figures and Tables

**Table 1:** Summary of the included studies in this meta-analysis.

Author	Year	Region	Duration	Sample size	Disease	Age	Method	Cut-off value	Survival	Follow-up, mo	NOS
Chen CN	2006	Taiwan	1995 - 1999	79	GC	Mean 63.3	IHC	positive cells > 10%	OS	3 - 46	8
Yamaguchi K	2005	Japan	1976 - 1995	135	GC	Mean 60.6	IHC	positive cells ≥ 25%	OS	NR	8
Glynn SA	2010	USA	1993 - 2003	248	breast cancer	Mean 55.0	IHC	NR	CSS	NR	7
Wang, J	2016	USA	NR	107	PC	Mean 67.1	IHC	median	OS	NR	7
Zafirellis K	2010	Greece	1991 - 2000	132	CRC	Mean 70.0	IHC	score ≥ 3	CSS	85 - 200	8
Zhang W	2011	China	1999 - 2004	211	GC	Mean 59.0	IHC	score ≥ 4	OS	≥ 60	9
Wang RH	2018	China	NR	90	HCC	NR	IHC	> 2 fold mean expression of normal tissue	OS	NR	7
Johansson CC	2010	Sweden	NR	90	uveal melanoma	Mean 62.0	IHC	positive cells ≥ 10%	CSS	1.7 - 262.8	8
Raspollini MR	2004	Italy	1985 - 1999	78	OC	Mean 58.0	IHC	positive cells ≥ 10%	OS, RFS	3 - 204	7
Ekmekcioglu S	2006	USA	1994 - 1997	132	melanoma	Median 51.0	IHC	positive cells ≥ 5%	CSS	Median 49.5	8
Garrido P	2017	Ireland	1999 - 2015	209	bladder cancer	Median 57.0	IHC	score ≥ 2	CSS	NR	7
Pukkila MJ	2002	Finland	NR	118	HSCC	Mean 64.0	IHC	score ≥ 4	OS	Median 43	7
Shigyo H	2007	Japan	1990 - 2001	63	LSCC	Median 67.0	IHC	score ≥ 3	RFS	Median 39	7
Li LG	2005	China	NR	66	GC	Mean 57.0	IHC	score ≥ 5	OS	NR	7

NR: not reported; GC: gastric cancer; PC: pancreatic cancer; CRC: colorectal cancer; HCC: hepatocellular carcinoma; OC: ovarian cancer; HSCC: hypopharyngeal squamous cell carcinoma; LSCC: laryngeal squamous cell carcinoma; IHC: immunohistochemistry; OS: overall survival; CSS: cancer-specific cancer; RFS: recurrence-free cancer; NOS: Newcastle-Ottawa Scale.
